# Understanding Detrimental Aspects of Social Media Use: Will the Real Culprits Please Stand Up?

**DOI:** 10.3389/fsoc.2020.599270

**Published:** 2020-12-01

**Authors:** Christian Montag, Simon Hegelich

**Affiliations:** ^1^Department of Molecular Psychology, Institute of Psychology and Education, Ulm University, Ulm, Germany; ^2^Political Data Science, Technical University of Munich, Munich, Germany

**Keywords:** Facebook, Instagram, personality, data business model, social media use, social media addiction, problematic social media use, social networks use disorder

## Much research investigating detrimental aspects of social media Use focuses on person characteristics and happens in isolation from each other

Currently, nearly four billion people all over the world use platforms such as Facebook, Instagram, WeChat or TikTok (Clement, 2020; Wearesocial.com, 2020). Given the impact that social media platforms have on humanity, it is not surprising that important lines of research seek to shed light on the *who* and *why* questions in the context of (over-)use of social media [for a comprehensive definition of the term “social media” see (Carr and Hayes, 2015)]. The *who*-question aims to understand *who* uses social media, whereas the *why*-question asks *why* people are using social media.

The *who*-question has been answered, among others, by personality psychologists, providing insights into sociodemographic variables and personality traits more likely being associated with social media use (Correa et al., 2010). A new large-scale study by Marengo et al. (2020) recently observed that social media users differ from non-users, with users (of Facebook-owned platforms) being younger, more often female and slightly more extraverted than non-users. Works such as by Brailovskaia and Margraf (2017) and Sindermann et al. (2020a,b), yielded insights that certain personality traits, such as being more neurotic/narcissistic, are associated with higher tendencies toward *problematic social media use* or *social networks use disorder*, with the terminology itself being a matter of fierce debate among scientists (Hussain et al., 2020; Wegmann et al., 2020; Montag et al., in press).

**Figure 1:**
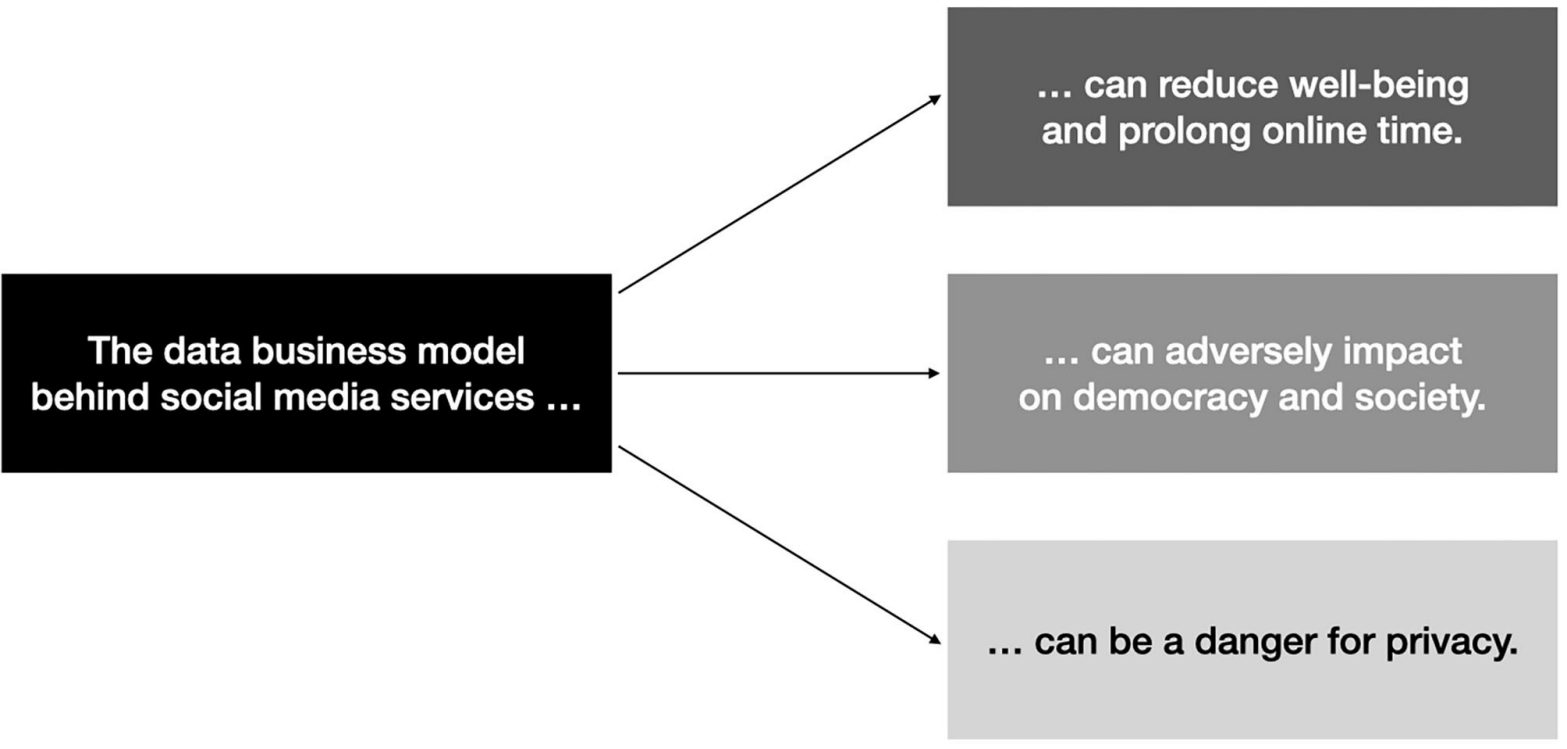
Detrimental aspects of the data business model behind social media services.

Of importance, *problematic social media use*—in light of a mental health problem—does not present the only detrimental aspect when (over-)using social media. Further detrimental aspects of social media use comprise misinformation campaigns via social media and loss of privacy for billions of humans. We are aware that valuable research exists in each mentioned area (e.g., Krasnova et al., 2010; Flaxman et al., 2016), but we believe that many researchers in their respective research fields under-estimate or at least under-emphasize that problems such as the addictive nature of social media, loss of privacy or problems arising for society due to misinformation campaigns in filter-bubbles all can indirectly be linked to each other – via the data business model behind social media platforms (see also Figure 1). This said, the importance to not investigate the aforementioned problems in isolation have been also put forward by laudable initiatives such as from the Center for Humane Technology (https://www.humanetech.com) stating on their website “As long as social media companies profit from outrage, confusion, addiction, and depression, our well-being and democracy will continue to be at risk.”

Against this background, it is not our objective in the present work to discuss the actual nature of excessive social media use, but rather to highlight the need to seek a new perspective on the prevalent research agenda, namely to keep in mind when studying detrimental aspects of social media use that the mentioned adverse aspects of social media use could be all solved to some extent when the data business model behind social media platforms would be improved or exchanged by a better alternative.

The fact that the distinct views taken by many scientists in their respective disciplines provide a too narrow view on the topic, for instance can be supported by empirical evidence: Coming back to the initially posed question “*who* uses social media?,” abundant evidence exists linking certain personality traits to excessive or problematic social media use, but effect sizes are usually only small to moderate. To illustrate this: Sindermann et al. (2020a) observed a small correlation of *rho* = 0.17 between the personality trait of neuroticism and Facebook use disorder leaving much room for other explanations on *who* spends (too much) time on social media. Of interest, similar observations regarding effect sizes can be made, when it has been investigated which personality traits are associated with daily news consumption via-social media only (Sindermann et al., 2020c) or understanding privacy concerns from a personality psychologist's perspective (Bansal et al., 2016).

Aside from the *who*-question, which pointed toward the personality structure being the culprit behind (over-)use of social media, *uses and gratification theory* carved out hedonic, utilitarian and social motives as highly relevant to understand *why* people (over-)use social media (Hsiao et al., 2016; Kircaburun et al., 2020). Ergo, *uses and gratification theory* tries to understand the *why*-question by investigating which basic human needs are satisfied by social media use. Again, correlations in this area usually also do not reach high effect sizes, leaving again much room for another important factor or factors driving detrimental aspects of social media use.

In sum, investigating personality traits or usage-motives in the context of detrimental aspects of social media use is helpful, but by far not enough to get the full picture of how to understand and tackle the manifold adverse aspects of social media (over-)use. We believe that the “real” culprit to be focused upon in research represents *the data business model* behind social media platforms and we will outline that a stronger focus on the data business model and social media platform design is needed in independent research.

## A Stronger Focus on the Data Business Model and Social Media Platform Design is Needed in Independent Research

As nothing comes free in life, we should not be surprised that we pay for the usage of a social media service with our personal data on a daily basis. Such a focus on harvesting digital footprints from each user to get better insights into their psychological profiles and to sell these insights to marketing companies (Matz et al., 2017; Azucar et al., 2018; Marengo and Montag, 2020) led engineers behind social media platforms to design applications which naturally aim at the prolongation of usage time. Longer social media usage equals more data on a user, and worsens the already excessive intrusion on individual privacy. In recent years, platforms like Facebook and YouTube have went from “more time spent” to “time well spent”: instead of optimizing the pure amount of time, platform-algorithms now try to show users content that triggers reactions in the form of “comments,” “shares” or “likes” (Papakyriakopoulos et al., 2020b). This might lead to even more time spent as well as to a more detailed digital footprint of the users (less privacy). Elements such as “Likes,” personalized news-feeds, endless scrolling, read receipts, to name a few, likely lead to more immersion on the user side (Montag et al., 2019). This keeps users longer on the social media platforms and/or lures them to check in more often than they like (see also push-notifications). Although the effects of the Like-button have been well-studied from a psychological point of view (Steinfield et al., 2008; Scissors et al., 2016; Burrow and Rainone, 2017; Zell and Moeller, 2018), the remaining in-built elements of social-media-platforms are understudied (e.g., the read-receipt, see a work by Blabst and Diefenbach, 2017). Furthermore, it is vastly understudied how each of these elements by themselves or in their interaction with each other actually drive human behavior on social media platforms and prolong the usage time. It is of utmost importance to get insights into effect sizes in this context. In addition, while the diversity of social-media-platforms is high, the cross-platform validity of an observed effect is questionable: the effects of a “Like” on Instagram cannot be simply transferred to TikTok (Serrano et al., 2020). On a methodological level, studying social media is challenging as well: Given an uneven distribution of activities—most users are quite passive in their usage while a handful of others produce the main share of reactions (Papakyriakopoulos et al., 2020b) —mean-related statistical measures are often misleading and analyzing these “non-normal” distributions requires huge datasets.

Social media platforms are, in many ways, black-boxes, where independent researchers looking from outside-in are handicapped by a variety of problems. The social media companies themselves possess richer data and insights into user behavior gained via AB-testing over many years. They know what combination of design-elements on social media platforms (also in Freemium-games on smartphones) function best in attracting and keeping the attention of their users. They also possess better insights into human behavior guided by design elements such as a personalized news-feed, which is widely believed to be responsible for filter-bubbles (Pariser, 2011) and echo-chambers (Shahrezaye et al., 2019), but likely only in the case when users inform themselves about the daily political news exclusively via social media (Sindermann et al., 2020c). Of note, personalized news-feeds are a good example to demonstrate how the addictive nature of social media and detrimental effects for society can be all linked to each other via a design-element and the data business model on social media. The personalized news-feed has been designed to create a highly interesting personalized website, where users like to spend much time and as a consequence produce more digital footprints (imagine the contrary: a boring news-feed would result in lower online time). On the one side this design element leads to higher online-time with higher risk for users to develop addictive tendencies toward the platform. On the other side this design element (fulfilling its purpose of the data collection) can result in filter-bubbles, because the social media companies typically show users what they are likely interested in (e.g., by having “liked” something earlier on social media).

With these few examples, it becomes apparent, that research on social media usage not only touches upon issues related to individual well-being and health, but also has broad political and privacy dimensions (see also Figure 1).

## Outlook

**Figure 2:**
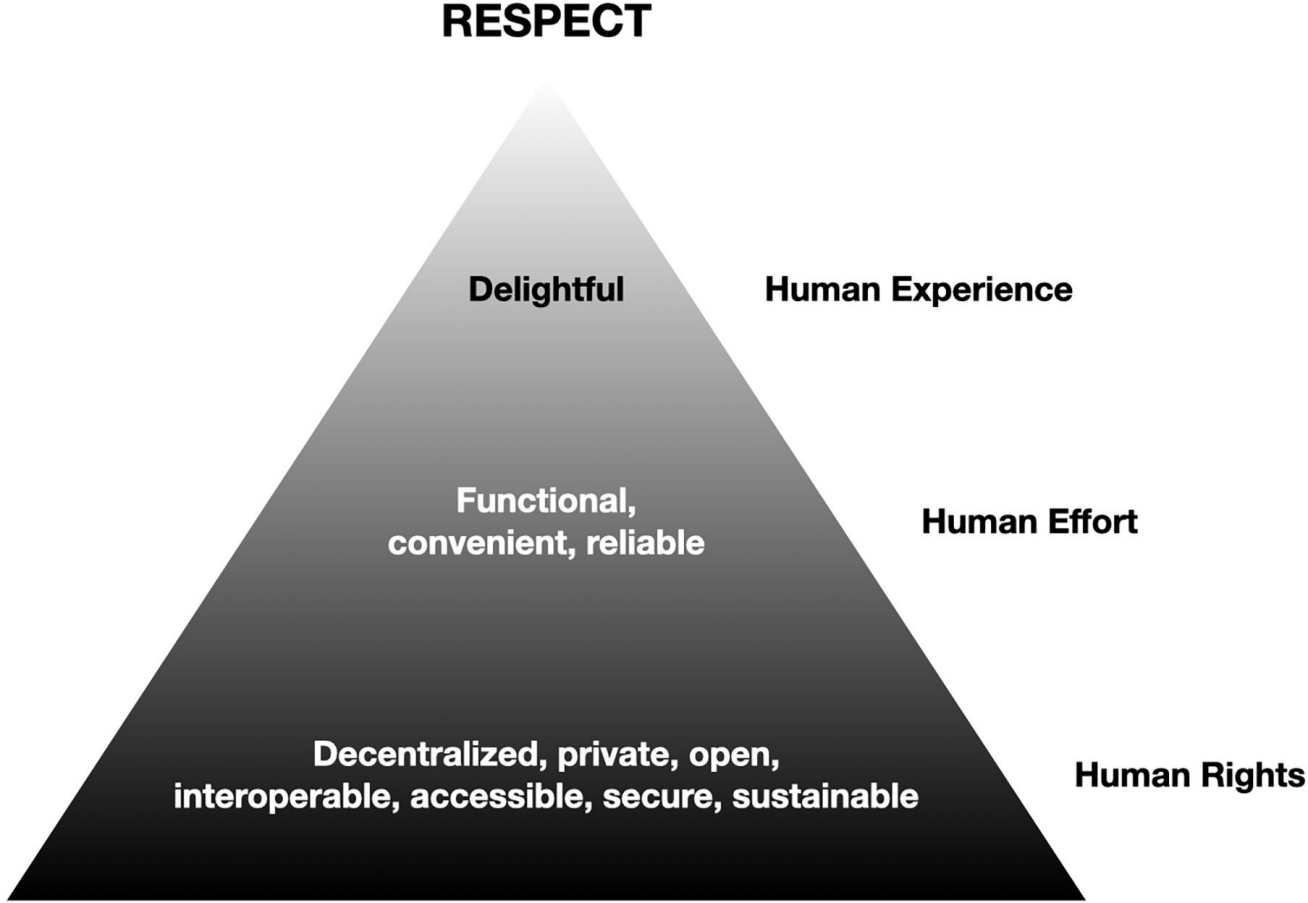
The *ethical design manifesto* according to the initiative of Ind.ie (https://2017.ind.ie/ethical-design/). The pyramid is presented in original wording as presented on the Ind.ie website. The graphical presentation slightly differs. Please note that the figure is under Creative Commons Attribution 4.0 International.

In order to be able to establish sustainable guidelines and policies toward social media platforms that do not aim to prolong online time (addictive potential of social media), and to protect the individual from (potentially) detrimental aspects of social media use, such as being caught in the filter-bubble (Sindermann et al., 2020c) and loss of privacy (Zuboff, 2015), it is of importance to (a) rethink the data business model (Sindermann et al., 2020d) and (b) to understand exactly what a “good” or “healthy” social media platform might look like. It is of particular relevance that academic research and public policy work toward building of a social media architecture that does not endanger democratic processes (Shahrezaye et al., 2019; Papakyriakopoulos et al., 2020b) or fosters sexism (Papakyriakopoulos et al., 2020a), radicalization or “fake news” and conspiracy theories (Papakyriakopoulos et al., 2020b). For example, the aforementioned problems around filter-bubbles and echo-chambers might be mitigated by excluding domains such as daily political news from news-feed personalization. Also, for instance, hiding “Likes” from users might diminish problems linked to social comparison and undue reinforcement of social media usage habits. An easy to understand orientation concerning healthy and fair social media platform design represents the *ethical design manifesto* (https://2017.ind.ie/ethical-design/; see also Figure 2). Using a Maslow like pyramid three stages are proposed – all to be considered to create fair online platforms. The bottom of the pyramid asks engineers to build among others decentralized, secure, sustainable and open platforms respecting human rights. One step higher in the pyramid it is argued to design platforms which are functional, convenient and reliable. Respecting these design principles ensures that humans do not waste life energy while visiting and interacting with an online platform (human effort should be taken into account). Finally, the persons behind the initiative value human experience, in short - the interaction with the online platform should be a fun experience.

We believe this *ethical design manifesto* to be of interest, but both the manifesto itself together with our earlier ideas need extensive testing for the validity of their premise and the relative merits of the various forms in which they could be implemented. Therefore, it is of utmost importance to get access to real-world data from tech-companies such as Facebook to answer such questions [but see problems with the recently launched “Social Science One”-initiative; (Ledford, 2019; Hegelich, 2020)]. Alternatively, when it is not possible to web-scrape data without intruding into user privacy, one could invest more research energy and funding into simulations of social media platforms, where different constellations of in-built features are tested for a variety of research questions targeting usage time, well-being (Brooks, 2015; Duradoni et al., 2020) and effects of filter-bubbles on radicalization (Zuiderveen Borgesius et al., 2016), among others. Additionally, it is crucial to combine information on digital footprints with self-report data in order to get deeper insights into *how* different groups of people are using social media (Montag and Elhai, 2019; Peterka-Bonetta et al., in press). Ultimately, the *who*-, *why*- and *how*- questions need to be brought together.

In sum, to tackle problems related to social media usage, it is high time to point to the real culprit: the data business model behind social media platforms and their design in itself. As also depicted in Figure 1, detrimental aspects of social media use can be seen in very different research areas, therefore scientists need also to reflect on this bird's eye view if they really want to change social media for the better, and this regardless on which area of social media research they are in. It needs to be mentioned that the different problem areas related to social media use likely impact differently on society in terms of persons afflicted. Whereas overuse of social media or being caught in the filter-bubble might only be a problem for a “few percent” of an investigated population at the moment (Bányai et al., 2017; Sindermann et al., 2020c; Wartberg et al., 2020), we are convinced that loss of privacy is a problem for every single user of a social media platform.

As the actual data business model has proven negative effects on society, it should be clear that more and more rigor and regulation is needed, just as it is for other forms of general infrastructure in society. Regulation could happen from the governmental side prohibiting the extent to which data is collected. Results from independent research could help to provide design-guidelines to answer what design-elements can be implemented (in what combination) on social media platforms. Such regulation and research is overdue and is expected to have a wide impact. One could, in fact, put a number to how many people stand to profit from a rigorous scientific agenda investigating social media – nearly 4 billion and rising.

## Author Contributions

CM drafted the first version of this opinion, which was critically revised by SH. Both authors contributed to the article and approved the submitted version.

## Conflict of Interest

The authors declare that the research was conducted in the absence of any commercial or financial relationships that could be construed as a potential conflict of interest. Nevertheless, for reasons of transparency CM mentions that he has received (to Ulm University and earlier University of Bonn) grants from agencies such as the German Research Foundation (DFG). CM has performed grant reviews for several agencies; has edited journal sections and articles; has given academic lectures in clinical or scientific venues or companies; and has generated books or book chapters for publishers of mental health texts. For some of these activities he received royalties, but never from the gaming or social media industry. CM mentions that he is part of a discussion circle (Digitalität und Verantwortung: https://about.fb.com/de/news/h/gespraechskreis-digitalitaet-und-verantwortung/) debating ethical questions linked to social media, digitalization and society/democracy at Facebook. In this context, he receives no salary for his activities. Finally, he mentions that he currently functions as independent scientist on the scientific advisory board of the Nymphenburg group. This activity is financially compensated. The handling editor declared a past co-authorship with one of the authors, CM.
